# Supplementary material for: Spatial patterns and determinants of anxiety, depressive symptoms and their co-occurrence among currently married women of reproductive age in Bangladesh
Source: Glob Ment Health (Camb). 2025 Dec 22;13:e4. doi: 10.1017/gmh.2025.10121 (PMC12766536; doi:10.1017/gmh.2025.10121)
Supplement: Hossain et al. supplementary material [file S2054425125101210sup001.docx]

**Supplementary Materials**

**Supplementary Table 1.** Bivariate association between mental health symptoms and explanatory variables

| **Study variables** | Have Depressive symptoms | | | Have Anxiety Symptoms | | | CAD symptoms | | |
| --- | --- | --- | --- | --- | --- | --- | --- | --- | --- |
|  | No, n (%) | Yes, n (%) | p-value | No, n (%) | Yes, n (%) | p-value | No, n (%) | Yes, n (%) | p-value |
| Geographical region | | | <0.001 |  |  | 0.015 |  |  | 0.034 |
| Barishal | 721 (94.9) | 39 (5.1) |  | 728 (95.8) | 32 (4.2) |  | 742 (97.6) | 18 (2.4) |  |
| Chattogram | 1964 (96.2) | 78 (3.8) |  | 1953 (95.6) | 89 (4.4) |  | 1997 (97.8) | 46 (2.2) |  |
| Dhaka | 3389 (93.4) | 111 (6.6) |  | 3402 (96.4) | 126 (3.6) |  | 3465 (98.2) | 64 (1.8) |  |
| Khulna | 1559 (93.4) | 110 (6.6) |  | 1578 (94.5) | 92 (5.5) |  | 1614 (96.7) | 56 (3.3) |  |
| Mymensingh | 989 (96.7) | 34 (3.3) |  | 992 (97.1) | 31 (2.9) |  | 1011 (98.8) | 13 (1.2) |  |
| Rajshahi | 1900 (96.8) | 63 (3.2) |  | 1911 (97.3) | 52 (2.7) |  | 1932 (98.4) | 32 (1.6) |  |
| Rangpur | 1522 (92.1) | 131 (7.9) |  | 1566 (94.7) | 87 (5.3) |  | 1608 (97.3) | 45 (2.7) |  |
| Sylhet | 691 (93.7) | 46 (6.3) |  | 701 (95.1) | 36 (4.9) |  | 712 (96.6) | 26 (3.4) |  |
| Mass Media exposure | | | 0.421 |  |  | 0.499 |  |  | 0.819 |
| No | 5264 (95.4) | 252 (4.6) |  | 5283 (95.8) | 233 (4.2) |  | 5393 (97.8) | 124 (2.2) |  |
| Yes | 7469 (95.1) | 387(4.9) |  | 7545 (96.1) | 311 (3.9) |  | 7685 (97.8) | 172 (2.2) |  |
| Wealth index | | | 0.234 |  |  | 0.249 |  |  | 0.035 |
| Poorest | 2307 (94.8) | 127 (5.2) |  | 2323 (95.4) | 111 (4.6) |  | 2372 (97.4) | 63 (2.6) |  |
| Poorer | 2568 (95.1) | 131 (4.9) |  | 2597 (96.2) | 102 (3.8) |  | 2651 (98.2) | 49 (1.8) |  |
| Middle | 2643 (94.8) | 145(5.2) |  | 2675 (95.9) | 113 (4.1) |  | 2717 (97.4) | 72 (2.6) |  |
| Richer | 2660 (95.1) | 136 (4.9) |  | 2666 (95.3) | 129 (4.7) |  | 2722 (97.4) | 74 (2.6) |  |
| Richest | 2555 (96.2) | 100 (3.8) |  | 2567 (96.7) | 88 (3.3) |  | 2617 (98.5) | 39 (1.5) |  |
| Household members (numbers) | | | 0.073 |  |  | 0.129 |  |  | 0.208 |
| ≤4 | 6210 (94.8) | 336(5.2) |  | 6262 (95.7) | 285 (4.3) |  | 6391 (97.6) | 157 (2.4) |  |
| ≥5 | 6522 (95.6) | 303 (4.4) |  | 6567 (96.2) | 258 (3.8) |  | 6689 (97.9) | 139 (2.1) |  |
| Intimate partner violence | | | <0.001 |  |  | 0.002 |  |  | 0.012 |
| Low | 10788 (95.7) | 489 (4.3) |  | 10846 (96.1) | 432 (3.9) |  | 11045 (97.9) | 233 (2.1) |  |
| High | 1944 (92.9) | 150 (7.1) |  | 1983 (94.7) | 112 (5.3) |  | 2032 (97.1) | 63 (2.9) |  |
| Age (years) | | | <0.001 |  |  | <0.001 |  |  | <0.001 |
| 15-24 | 2698 (96.9) | 84 (3.2) |  | 2731 (98.1) | 51 (1.9) |  | 2755 (99.0) | 28 (1.0) |  |
| 25-34 | 4025 (95.1) | 208 (4.9) |  | 4077 (96.3) | 156 (3.7) |  | 4144 (97.9) | 90 (2.1) |  |
| 35-44 | 4388 (95.1) | 230 (4.9) |  | 4388 (95) | 230 (5) |  | 4508 (97.6) | 111 (2.4) |  |
| ≥45 | 1622 (93.2) | 1171 (6.8) |  | 1632 (93.9) | 106 (6.1) |  | 1671 (96.1) | 68 (3.9) |  |
| Education level | | | 0.013 |  |  | <0.001 |  |  | <0.001 |
| Less than high school | 3576 (95.1) | 185 (4.9) |  | 3588 (95.4) | 173 (4.6) |  | 3662 (97.4) | 100 (2.6) |  |
| High school | 5533 (95.6) | 259 (4.4) |  | 5584 (96.4) | 208 (3.6) |  | 5677 (98.0) | 116 (2.0) |  |
| More than high school | 1636 (96.9) | 53 (3.1) |  | 1650 (97.8) | 37 (2.2) |  | 1674 (99.1) | 15 (0.9) |  |
| Occupation | | | 0.061 |  |  | 0.001 |  |  | 0.110 |
| Homemakers | 10683 (95.3) | 520 (4.7) |  | 10776 (96.1) | 427 (3.9) |  | 10969 (97.9) | 236 (2.1) |  |
| Business | 843 (94.8) | 46 (5.2) |  | 844 (94.9) | 45 (5.1) |  | 867 (97.5) | 23 (2.5) |  |
| Service holder | 291 (97.3) | 8 (2.7) |  | 293 (97.9) | 6 (2.12) |  | 296 (98.8) | 4 (1.2) |  |
| Laborer | 916 (93.4) | 64 (6.6) |  | 915 (93.3) | 65 (6.7) |  | 947 (96.5) | 34 (3.5) |  |
| Body mass index | | | 0.026 |  |  | 0.296 |  |  | 0.265 |
| Underweight | 578 (92.6) | 47 (7.4) |  | 592 (94.9) | 32 (5.1) |  | 604 (96.6) | 22 (3.4) |  |
| Normal weight | 3284 (95.2) | 162 (4.8) |  | 3312 (96.1) | 134 (3.9) |  | 3369 (97.7) | 79 (2.3) |  |
| Overweight | 2387 (95.1) | 124 (4.9) |  | 2396 (95.4) | 114 (4.6) |  | 2452 (97.7) | 58 (2.3) |  |
| Sexual inactivity | | | 0.008 |  |  | <0.001 |  |  | 0.008 |
| No | 11601 (95.4) | 549 (4.6) |  | 11696 (96.2) | 455 (3.8) |  | 11904 (97.9) | 247 (2.1) |  |
| Yes | 943 (92.8) | 73 (7.2) |  | 949 (93.4) | 67 (6.6) |  | 981 (96.6) | 35 (3.4) |  |
| Pregnancy termination | | | 0.073 |  |  | <0.001 |  |  | 0.044 |
| No | 9717 (95.4) | 466 (4.6) |  | 9814 (96.3) | 369 (3.7) |  | 9975 (97.9) | 209 (2.1) |  |
| Yes | 3016 (94.6) | 173 (5.4) |  | 3014 (94.5) | 175 (5.5) |  | 3103 (97.3) | 87 (2.7) |  |
| Children ever born | | | <0.001 |  |  | <0.001 |  |  | <0.001 |
| 1-2 | 6616 (96.1) | 275 (3.9) |  | 6667 (96.8) | 224 (3.2) |  | 6774 (98.3) | 118 (1.7) |  |
| 3-4 | 3892 (94.2) | 239 (5.8) |  | 3930 (95.1) | 201 (4.9) |  | 4014 (97.2) | 118 (2.8) |  |
| ≥5 | 837 (93.3) | 59 (6.7) |  | 823 (91.9) | 73 (8.1) |  | 861 (96.0) | 36 (4.0) |  |
| Husband's current age (years) | | | <0.001 |  |  | <0.001 |  |  | <0.001 |
| <40 | 5529 (96.1) | 227 (3.9) |  | 5610 (97.4) | 146 (2.6) |  | 5677 (98.6) | 81 (1.4) |  |
| 40-49 | 3898 (95.3) | 195 (4.7) |  | 3908 (95.4) | 185 (4.6) |  | 3988 (97.4) | 106 (2.6) |  |
| 50-59 | 2530 (94.1) | 161 (5.9) |  | 2540 (94.4) | 151 (5.6) |  | 2618 (97.3) | 74 (2.7) |  |
| ≥60 | 775 (93.3) | 55 (6.7) |  | 769 (92.6) | 62 (7.4) |  | 796 (95.7) | 36 (4.3) |  |
| Husband's occupation | | | <0.001 |  |  | <0.001 |  |  | <0.001 |
| Unemployed | 461 (91.2) | 44 (8.8) |  | 463 (91.6) | 43 (8.4) |  | 474 (93.8) | 32 (6.2) |  |
| Farmer/ laborer | 6786 (95.4) | 326 (4.6) |  | 6820 (95.9) | 292 (4.1) |  | 6969 (98.0) | 142 (2.0) |  |
| Business | 4534 (95.1) | 237 (4.9) |  | 4590 (96.2) | 181 (3.8) |  | 4668 (97.8) | 104 (2.2) |  |
| Service Holder | 941 (96.9) | 31 (3.1) |  | 945 (97.2) | 27 (2.8) |  | 955 (97.2) | 18 (1.8) |  |

**
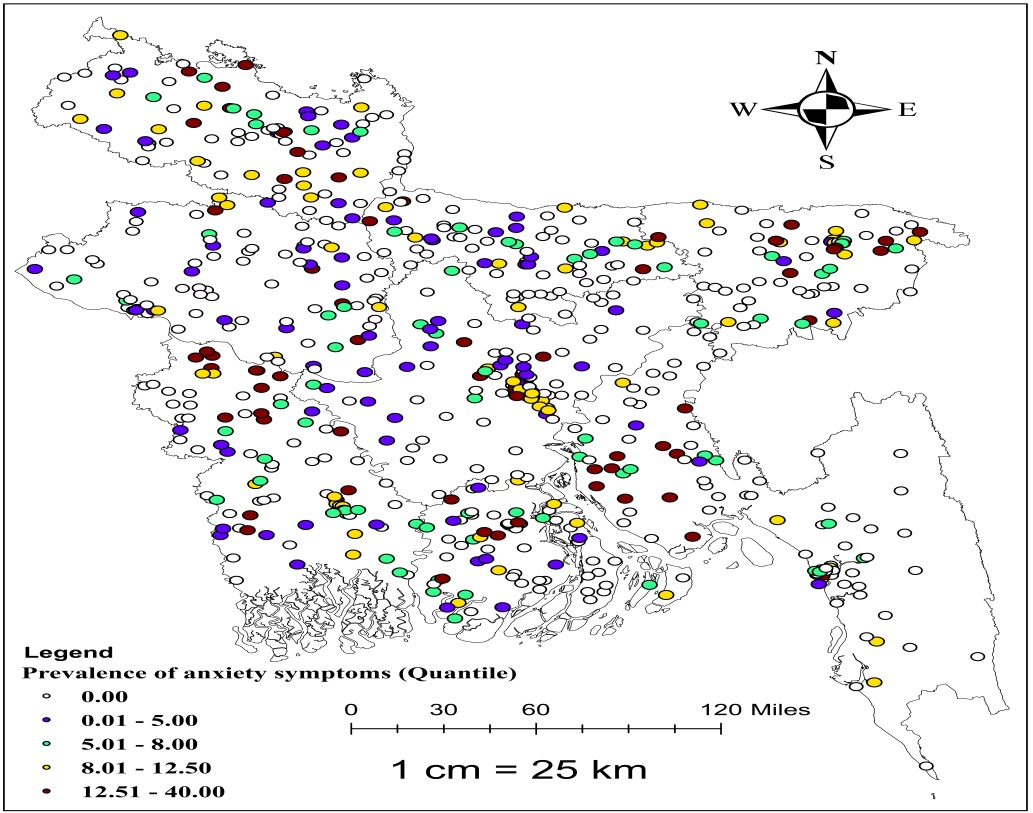
**

**Supplementary Figure 1.** Spatial distribution of anxiety symptoms among reproductive-aged married women in Bangladesh

**
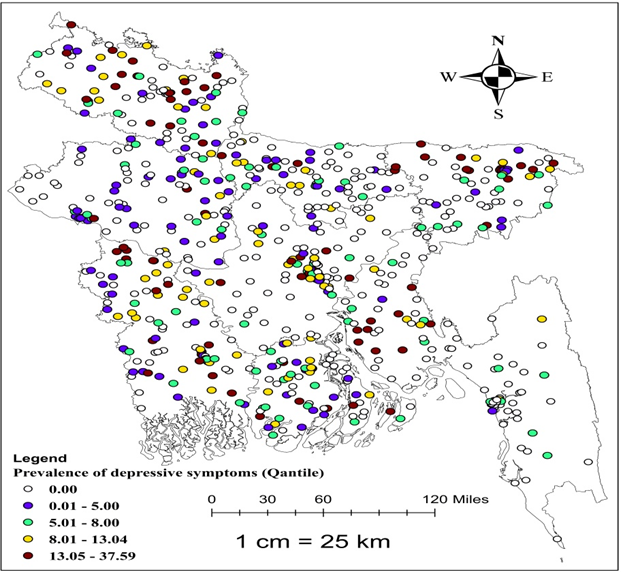
**

**Supplementary Figure 2.** Spatial distribution of depressive symptoms among reproductive-aged married women in Bangladesh

**
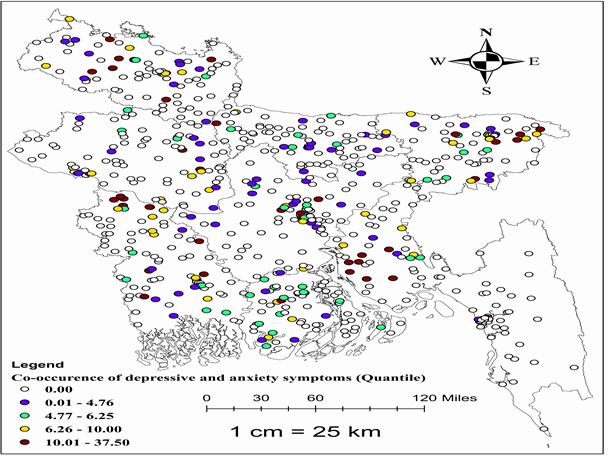
**

**Supplementary Figure 3.** Spatial distribution of the CAD symptoms among reproductive-aged married women in Bangladesh
